# Electrosprayed Core–Shell Composite Microbeads Based on Pectin-Arabinoxylans for Insulin Carrying: Aggregation and Size Dispersion Control

**DOI:** 10.3390/polym10020108

**Published:** 2018-01-23

**Authors:** Agustín Rascón-Chu, Jonathan A. Díaz-Baca, Elizabeth Carvajal-Millan, Elías Pérez-López, Arland T. Hotchkiss, Humberto González-Ríos, Rene Balandrán-Quintana, Alma C. Campa-Mada

**Affiliations:** 1 Research Center for Food and Development, CIAD, A.C., Carretera a La Victoria Km. 0.6, Hermosillo, Sonora 83304, Mexico; mcjonathandiaz@gmail.com (J.A.D.-B.); ecarvajal@ciad.mx (E.C.-M.), hugory@ciad.mx (H.G.-R.); rbalandran@ciad.mx (R.B.-Q.); acampa@ciad.mx (A.C.C.-M.); 2 Institute of Physics, Autonomous University of San Luis Potosí, Álvaro Obregón #64, San Luis Potosí SLP 78000, Mexico; elias@ifisica.uaslp.mx; 3 Agricultural Research Service, Eastern Regional Research Center, 600 E. Mermaid Lane, Wyndoor, PA 19038, USA; arland.hotchkiss@ars.usda.gov

**Keywords:** pectin, arabinoxylan, insulin microbeads, electrospray, core–shell composite

## Abstract

Aggregation and coalescence are major drawbacks that contribute to polydispersity in microparticles and nanoparticles fabricated from diverse biopolymers. This study presents the evaluation of a novel method for the direct, electrospray-induced fabrication of small, CaCl_2_/ethanol-hardened low methoxy pectin/arabinoxylans composite microbeads. The electrospray method was evaluated to control particle size by adjusting voltage, flux, and crosslinking solution content of CaCl_2_/ethanol. A bead diameter of 1µm was set as reference to test the capability of this method. Insulin was chosen as a model carried molecule. Statistical analysis was a central composite rotatable design (CCRD) with a factorial arrangement of 2^4^. The variables studied were magnitude and particle size dispersion. For the determination of these variables, light diffraction techniques, scanning electron microscopy, transmission electron microscopy, and confocal laser scanning microscopy were used. Major interaction was found for ethanol and CaCl_2_ as well as flow and voltage. Stable spherical structures of core–shell beads were obtained with neither aggregation nor coalescence for all treatments where ethanol was included in the crosslinking solution, and the average diameter within 1 ± 0.024 μm for 11 KV, 75% ethanol with 11% CaCl_2_, and flow of 0.97 mL/h.

## 1. Introduction

Polysaccharides are widely used as protectors and carriers and are generally considered non-toxic and biocompatible materials. Pectin and arabinoxylans have been proposed and studied as matrices for oral delivery of drugs and bioactives, due to their gelling capacity and biodegradability in the colon [1,2,3,4]. Pectin are components of plant cell walls, contribute to their rigidity springiness, and are key to cell growth regulation [5,6,7]. The gelling mechanism of pectin depends on the degree of esterification of the galacturonic acid contained in the pectin chain [8]. Low degree of esterification (LDE) pectin gels in the presence of divalent ions (i.e., Ca^2+^) forming a structure called an “egg box” [9]. This mechanism is a fast process, which provides stability and strength to the gel, tough sensitive to pH [10]. Arabinoxylans are commonly found in the cell wall of Poaceae species, like grasses and gramineae [11]. The polymeric chain consists of xylose in β-1,4 with ramifications of α-l-arabinofuranose in α-1,3 and α-1,2. In ferulated arabinoxylans (AXF), arabinose monomers exhibit ferulic acid residues [12]. AXF gels are formed by oxidative coupling of ferulic residues (mediated by oxidative agents, like laccases), producing a covalent gel that is stable to pH and temperature changes. Nevertheless, this process is slow, which impairs the stabilization of the structures [13,14]. Therefore, in this work, we to complement the slow formation of AXF gels with rapid gelling LDE pectin. For fabrication purposes, the latter aimed to stabilize the particles and prevent the formation of aggregates, while the former provides resistance to acidic pH in the gastrointestinal tract, and both degrade in the colon. Insulin was chosen as a model carried molecule. To regulate the internalization and transport across the intestinal mucosa barrier, the particle size, composition, surface properties, and geometry of the particles are critical [15,16,17]. Particularly, size in insulin-loaded particles is very important for bioavailability of the said carried insulin [18]. Based on previous studies [15,16,17,18], and according to the capacity of the manufacturing method, an optimum particle size of 1 μm was targeted.

There is a wide variety of methods for carrier manufacture, but for our goal, electrospray is very attractive. Electrospray involves the application of an electrodynamic force on a controlled drip flow and is characterized as a fast, efficient, and non-destructive method [19,20,21]. Electrospray has been used with various synthetic and natural polymers [22,23,24] and for subcutaneous-intended insulin nanoparticles, preserving intact biological activity [25].

Electrospray system with a coaxial flow allows us to jointly apply LDE pectin and AXF without mixing them, exploiting the properties of each polysaccharide to fabricate core shell–type microbeads as insulin carriers [26]. 

## 2. Materials and Methods

### 2.1. Polysaccharides and Gelling Agents

Pectin was extracted from thinning apple (*Malus domestica* Borkh.) with a degree of esterification of 41% (TA pectin). The AXF from pericarp of maize (*Zea mays*) was extracted and characterized as reported previously (A/X ratio of 0.85, AF = 0.34 μg/mg AXF, and an intrinsic viscosity of 208 mL/g) [27]. As carried molecule, human recombinant insulin (SAFC-91077C-SIGMA) was used. Laccase (Sigma-Aldrich 53739, St. Louis, MO, USA) and calcium chloride (Sigma-Aldrich C50080) were used as crosslinking agent for AX and pectin, respectively.

### 2.2. Modification of Pectin Esterification Degree

TA pectin was subjected to chemical treatment to reduce the esterification degree. Alkaline treatment was carried out for 2 h, with a 2 M NaOH (1 g pectin/mL alkaline solution) (Chemicals Monterrey SA, Ciudad de México, Mexico, 36904), under dark conditions. The reaction was stopped with cold (4 °C) 4 M HCl solution (Sigma-Aldrich 258148) and adjusted at pH 2.2. Subsequently, cold ethanol (4 °C) was added to a final ratio of 70% ethanol, mixed and stored under refrigeration for 24 h. The ethanolic solution was filtered with vacuum and glass fiber filters (Whatman™, Maidstone, UK, 1825-047). The sediment on the filter was dried in an oven at 60 °C for 12 h and then powdered.

The final degree of esterification in deesterified (DTA) pectin was determined by infrared spectroscopy on a FT-IR Nicolet Protege 460^®^ (Thermo Scientific, Waltham, MA, USA) according to the method described by Urias-Orona et al. [28].

### 2.3. Polysaccharides Dispersions

DTA pectin dispersion was prepared at 1% (*w*/*v*) in double distilled water and left under stirring for 24 h to ensure maximum dispersion. AXF dispersion was prepared at 6% (*w*/*v*) in a buffer solution (acetic acid/sodium acetate, 300 mM at pH 5.5) under constant stirring for 24 h. Once both polysaccharides dispersions were obtained, they were filtered through a metal syringe filter (steel mesh, hole size 350 μm).

### 2.4. Insulin Solubilization

This process was performed according to previous work by Martínez-López et al. [29]. An insulin solution was prepared (3.6 mg/mL). The insulin solution was mixed with AXF (6%) in a ratio of 1:1 (*v*/*v*) at a final concentration of 3% AXF (*w*/*v*). 

### 2.5. Electrospray Process

**Figure 1:**
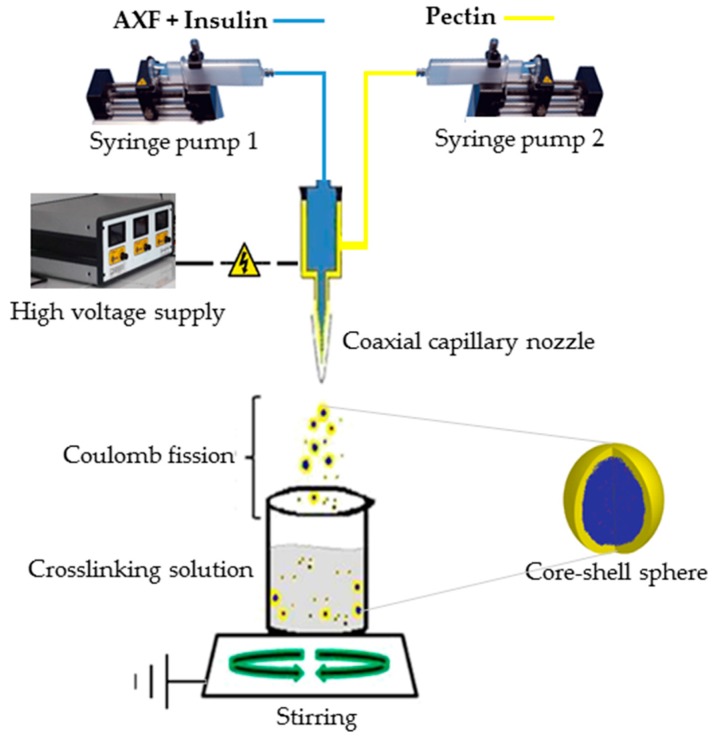
Diagram of coaxial-electrospray system used to produce pectin/arabinoxylan beads. (Adapted from Díaz-Baca et al., 2014 [26]).

A coaxial-electrospray Spraybase™ system (Profector™, Dublin, Ireland) and two programmable syringe pumps (WorldPrecision Instruments, AL-1000, Sarasota FL, USA), independently connected to the coaxial metallic needle (Figure 1), were used. In order to receive spraying droplets, a crosslinking solution was employed containing CaCl_2_, water, and ethanol. Injection flow, applied voltage, and CaCl_2_ and ethanol concentration in the crosslinking solution were varied for each experimental run according to experimental design described in Table 1.

The syringe from the outside of the needle contained DTA pectin, and the AXF-Ins solution was in the central section of the needle, along with 1.25 U/mL of laccase. One hundred μL of each polysaccharide solution were injected. The reception of the spray was performed in a volume of 10 mL of crosslinking solution with constant stirring at 200 rpm (Corning^®^, PC-420D, Corning, NY, USA) during the manufacturing time and further stirring for 2 h as a gel curing period. Samples were stored at 4 °C.

### 2.6. Measurement of Beads Size by DLS

A measurement of bead size and its distribution were made using dynamic light scattering (DLS). The measurements were performed on a 2 mL sample in a Particle Size Analyzer (Delsa™ Nano C, BeckmanCoulter, Guangzhou, China). The incidence of the laser light was at 658 nm wavelength. The beads were analyzed in the original crosslinking solution in triplicate. The mean size values and coefficients of variation (CV) were used as size dispersion indexes for “bead population.”

### 2.7. Scanning Electron Microscopy Low Vacuum (SEM-LV)

Scanning electron microscopy (SEM) allows us to observe the beads’ morphology. Observations were performed on a JEOL scanning electron microscopy JSM-6610/LV30 KV (JEOL USA, Inc., Peabody, MA, USA) in low vacuum mode 20 KV with different voltage amplifications as required for each sample. Samples were putted on a silicon surface and were evaporated under 60 W incandescent lighting for 2 h prior to analysis.

### 2.8. Transmission Electron Microscopy

Transmission electron microscopy (TEM) was used to complement the results obtained by SEM. To analyze the samples in digital imaging, 10 μL samples were deposited on a copper cell coated with formvar-carbon (Electron Microscopy Sciences, Hatfield, PA, USA) using a JEOL JEM-1230, 120 KV high-contrast transmission electron microscope (JEOL USA, Inc.) with an accelerating voltage of 100 KV at magnifications of 4000× to 120,000×.

### 2.9. Confocal Laser Scanning Microscopy

Confocal Laser Scanning Microscopy (CLSM) was used to determine the spatial location of insulin within beads, and verify the formation of core–shell type structure. A confocal high-resolution system Leica TCS SPE B with an inverted microscope DMI4000 (Leica Microsystems, Wetzlar, Germany) was used for this purpose. Protein fluorescence was observed using excitation wavelengths of 635 nm and emission of 620–680 nm. Images were observed at magnifications of 40× and 100× (oil immersion).

### 2.10. Experimental Design

Particle size (PS) and dispersion of size (CV) were used as response variables for a central composite rotatable design (CCRD). The factorial arrangement 2^4^, two factorial levels (−1 and 1), a central level (0), and two axial levels (−α and α) are shown in Table 1. Factors studied and varied were injection flow *x*_1_, voltage *x*_2_, ethanol concentration *x*_3_, and CaCl_2_ concentration *x*_4_. A total of 28 randomized experimental runs were made with four replicates in central values to ensure repeatability and rotatability of the design.

Data from each experimental run were used to model the effect-related interaction between factors (*x*_1_, *x*_2_, *x*_3_, and *x*_4_) on particle size and its coefficient of variation. Data were analyzed by surface response methodology (SRM) to fit a second order polynomial model and explore the quadratic response surface. Design and analysis of data were performed in the statistical software JMP^®^ 11.1.0 (2013 SAS Institute Inc., Cary, NC, USA). The initial mathematical model (second-order polynomial) to correlate the responses (PS, CV) with the factors as follows (Equation (1)):

(1) $$\begin{array}{cc} Y=\;\beta_{0}+ & \beta_{1}\left(x_{1}\right)+\;\beta_{2}\left(x_{2}\right)+\beta_{3}\left(x_{3}\right)+\;\beta_{4}\left(x_{4}\right)+\;\beta_{11}\left(x_{1}\right){}^{2}+\;\beta_{22}\left(x_{2}\right){}^{2}+\;\beta_{33}\left(x_{3}\right){}^{2}\\ & +\;\beta_{44}\left(x_{4}\right){}^{2}+\;\beta_{12}\left(x_{1}\times x_{2}\right)+\;\beta_{13}\left(x_{1}\times x_{3}\right)+\beta_{14}\left(x_{1}\times x_{14}\right)+\beta_{23}\left(x_{2}\times x_{3}\right)\\ & +\beta_{24}\left(x_{2}\times x_{4}\right)+\beta_{34}\left(x_{3}\times x_{4}\right) \end{array}$$

where *Y* is a predicted response; *x*_1_, *x*_2_, *x*_3_, and *x*_4_ correspond to the value of independent variable; β_0_ is an intercept; β_1_, β_2_, β_3_, and β_4_ are linear regression coefficient; β_11_, β_22_, β_33_, and β_44_ are quadratic regression coefficient; and β_12_, β_13_, β_14_, β_23_, β_24_, and β_34_ correspond to the interaction regression coefficients. For our study, an optimum particle size of 1 μm was defined. For optimal values of factors, and analysis of interactions, predictive profile graphics and surface profile were performed only with significant parameters.

### 2.11. Fabrication Post-Optimization

To explore the effect on side dispersion of the statistical predictions, a further factorial design was carried out. The beads obtained under the new set of conditions were analyzed by SEM to assess their particle morphology and size.

### 2.12. Scanning Electron Microscopy (SEM)

For SEM analysis, 10 µL washed samples aliquots were placed on glass slides, and dried at room temperature using a steady flow of nitrogen for 2 h. The samples were coated by electroplating with gold and observed with a FEI Quanta 200F (FEI Company, Hillsboro, OR, USA) microscope at different magnifications, and an accelerating voltage of 10 KV. The images generated were analyzed with the open-domain software Image J2X (Rawak Software, Inc., Dresden, Germany). Ten fields per sample were observed randomly. NCSS 2007 was used for data analysis.

### 2.13. Sample Preparation for SEM Microscopy Analysis

Prior to analysis, the samples were “washed” in order to remove the medium present. The sample was filtered through a syringe filter steel mesh. The filtrate was discarded, and the residue on the filter was resuspended with 1 mL of ultrapure water. The samples were stored at 4 °C.

## 3. Results and Discussion

### 3.1. Degree of Esterification

**Figure 2:**
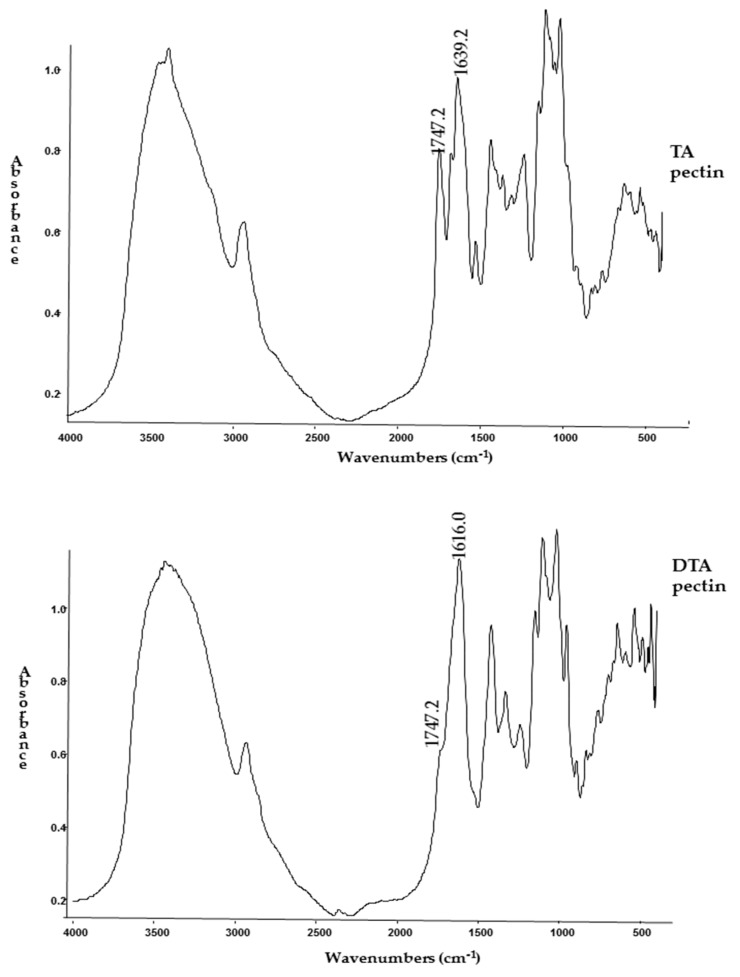
FT-IR absorption spectra for TA (untreated) pectin and deesterified (DTA) pectin (post treatment). The decrease in area of band absorbing at 1750 cm^−1^ on DTA pectin spectrum compared to TA pectin band absorbing at 1750 cm^−1^ evidence the decrease in esterified groups.

Alkaline hydrolysis enabled us to reduce the degree of esterification of the TA pectin, down on 86%, having a final DTA pectin of 5.6% ± 1.4% (mean ± standard error). The DTA and TA pectin absorption spectra are shown in Figure 2.

The production of beads by coaxial electrospray was carried out using DTA pectin solutions and AXF-insulin solution. The very low degree of esterification accelerated the nucleation process with calcium, creating an exterior “crust” stable enough to keep droplets from gelling together and maintain the spherical structure of the droplets formed in the electrospray. The latter allowed the AXF-insulin covalent crosslinking to take place at the core of each droplet independently.

### 3.2. Shape and Size of Beads

**Figure 3:**
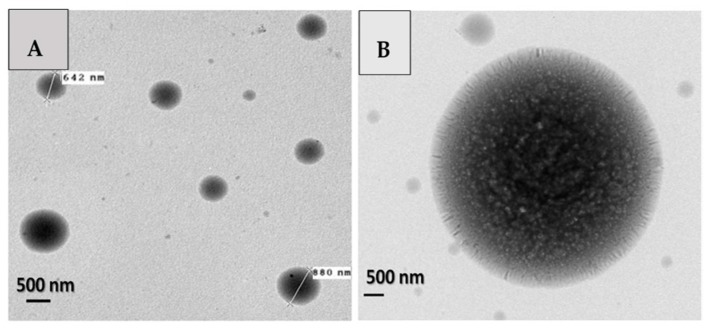
Examples of negative stain transmission electron microscopy (TEM) images of beads. Conditions of ethanol concentration and CaCl_2_ concentration are: (**A**) 0.163 mL/h, 14 KV, 87.5% ethanol, 6.5% CaCl_2_; (**B**) 0.163 mL/h, 14 KV; 100% ethanol, 15.5% CaCl_2_.

Average diameters of beads, obtained by DLS, were in the range of 0.58–28.89 μm, and 16 of the 28 experimental runs had average diameters lower than 3 μm. In four experimental runs, average diameters lower than 1 μm were found. Some representative microscopy images are shown in Figure 3. Moreover, the samples exhibited high rates of dispersion in diameter values, in the range of 3% to 55% CV. CaCl_2_ crystal nucleation was very important, and the authors assumed it originated the wide range of particles size. Core–shell arrangement of the particles and no aggregation was common to the treatments.

In the SEM-LV images, only the beads larger than ≈1 μm were observed, limited by the resolution capability of SEM in low vacuum mode. In these images, the geometry and spherical symmetry of the structures are remarkable as well as the stability of the beads’ individual structures. No evidence of aggregation was found. Due to resolution being larger than the aimed size, images are not shown; further SEM images confirm the results for stability of particle surface and shape.

In counterpart, in the TEM images (Figure 3), it was possible to observe structures smaller than ≈1 μm. The results of geometry and symmetry previously found in the SEM images are supported with the images obtained by TEM, which found similar results. In the images, a presumable structure of core–shell type for beads was found. In the central section of beads, a darker area compared to the outer section is observed; similarly, it appears to have a different composition and arrangement. Another relevant feature was found in these images: the existence of a kind of interface region between the central section and the outer section, possibly between DTA pectin and AXF-insulin.

**Figure 4:**
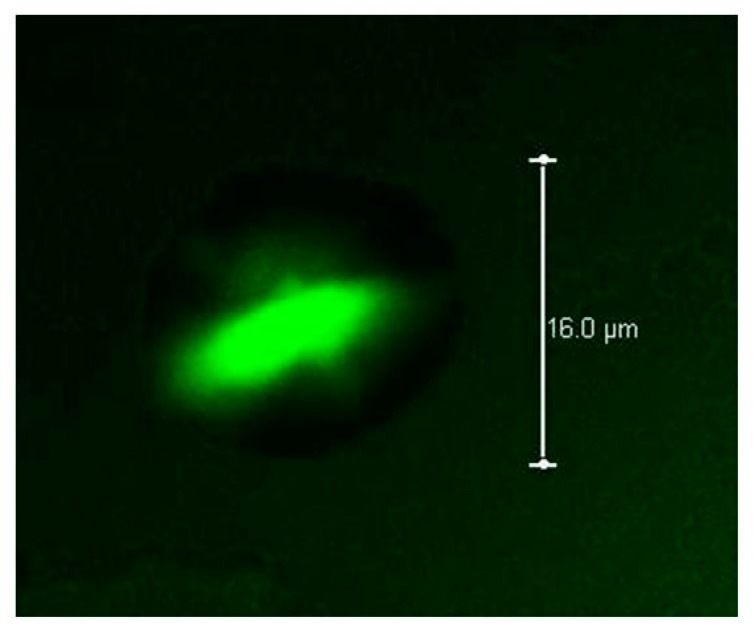
Confocal Laser Scanning Microscopy (CLSM) images. Protein autofluorescence showed in the center of beads (intense color). Conditions of injection flow, applied voltage, ethanol concentration, and CaCl_2_ concentration are 0.163 mL/h, 14 KV; 87.5% ethanol, 15.5% CaCl_2_.

In Figure 4, self-fluorescence of the proteins is observed. The beads exhibit fluorescence only in the central section, and a black tone in the surrounding outer section can be appreciated. With these results, it may be stated that the beads have a core containing proteins (mainly insulin) and a shell of polysaccharides (mostly DTA pectin in theory).

With the above mentioned, the combination of DTA pectin and AXF in a coaxial system allows the formation and stabilization of hydrogels beads. DTA pectin allowed keeping the spherical structure of the microdroplets formed in the electrospray, preventing aggregation of AXF-Insulin gels. Through its rapid gel formation mechanism, DTA pectin interact with Ca^2+^ molds and stabilizes the droplets, while AXF chemical gels, trapping insulin in the inner section of the beads to ‘’mature’’ and stabilize. At this stage, we can clearly see that protein is contained in the core of particles from different set of fabrication conditions.

Ghayempour and Mortazavi [30] worked with coaxial-electrospray systems previously, which included alginate (alginate has a gelling mechanism analogue to pectin). Accordingly, they found the formation of symmetrical and spherical structures with no evidence of aggregation. This indicates that the nucleation of calcium on the surface of the spheres with anionic polysaccharides promotes stabilization of the particles through ionic charges.

Furthermore, regarding beads size, it was found that both the diameter and the size dispersion varied in each experimental run. In a previous coaxial-electrospray study, Cao et al. [31] reported production of PLA–PEG spheres with a core–shell morphology and found an average diameter of about 3 μm. With this evidence, and the present work, it is conclusive that the coaxial-electrospray system is effective for manufacturing core–shell type microbeads based both on synthetic polymers and/or biopolymers, enabling the control of particle size.

In contrast to other studies [32,33] involving other methods of manufacture, the coaxial electrospray allowed us to locate insulin in the core of the beads and not dispersed in the entire structure. This arrangement benefits the conservation properties of insulin by having protective DTA pectin shell while being trapped in a covalent network of AXF. Overall, this “armor” should avoid premature insulin release and enzymatic degradation in the upper gastrointestinal tract. Nevertheless, this statement requires evidence on animal models. Further research in this regard is being carried out.

### 3.3. Statistical Analysis

The most suitable predictive model was sought based on the significance of the different parameters and the coefficient of determination (*R*^2^). The original model was adjusted, removing the parameters with the highest probability and with the minor *R*^2^ modification. Linear parameters were excluded from deletion due to intrinsic factors. 

In Equations (2) and (3), the fitted models for particle size and CV are shown. Particle size model presents a significance of *p* = 0.0102, with an acceptable predictive power of *R*^2^ = 0.731. Any of the linear factors were significant (*p* > 0.05), the same as quadratic parameters (*p* > 0.05).

(2) $$\begin{array}{cc} \mathrm{PS}= & -52.488-6770.748(\mathrm{flow})+397.411(\mathrm{voltage})\\ & -40.049(\mathrm{ethanol}\;\mathrm{Conc}.)+247.409(\mathrm{CaCl}{}_{2}\;\mathrm{Conc}.)-11623.54\\ & +2047.410(\mathrm{flow}\times \mathrm{ethanol}\;\mathrm{Conc}.)\\ & -120.480(\mathrm{voltage}\times \mathrm{ethanol}\;\mathrm{Conc}.)+8.714(\mathrm{ethanol}\;\mathrm{Conc}.){}^{2}\\ & -5474.928(\mathrm{flow}\times \mathrm{CaCl}{}_{2}\;\mathrm{Conc}.)+294.301(\mathrm{voltage}\times \mathrm{CaCl}{}_{2}\;\mathrm{Conc}.)\\ & -46.109(\mathrm{ethanol}\;\mathrm{Conc}.\times \mathrm{CaCl}{}_{2}\;\mathrm{Conc}.) \end{array}$$

(3) $$\begin{array}{cc} \mathrm{CV}=17.995 & +40.501(\mathrm{flow})+1.065(\mathrm{voltage})-0.288(\mathrm{ethanol}\;\mathrm{Conc}.)\\ & -0.482(\mathrm{CaCl}{}_{2}\;\mathrm{Conc}.)+127.964(\mathrm{flow}){}^{2}-11.914(\mathrm{flow}\times \mathrm{voltage})\\ & -0.094(\mathrm{voltage}\times \mathrm{ethanol}\;\mathrm{Conc}.)-0.012(\mathrm{ethanol}\;\mathrm{Conc}.){}^{2}\\ & -11.082(\mathrm{flow}\times \mathrm{CaCl}{}_{2}\;\mathrm{Conc}.)+0.456(\mathrm{voltage}\times \mathrm{CaCl}{}_{2}\;\mathrm{Conc}.)\\ & -0.060(\mathrm{ethanol}\;\mathrm{Conc}.\times \mathrm{CaCl}{}_{2}\;\mathrm{Conc}.)+0.481(\mathrm{CaCl}{}_{2}\;\mathrm{Conc}.){}^{2} \end{array}$$

The PS fitted model predicts that individual factors will not have significant influence on the particle size, and the interaction is necessary to achieve a prediction. Accordingly, predetermined particle size can be achieved by factors combined specifically and not by one of them solely.

For CV, the reduced model (Equation (3)) show a significance of *p* = 0.0059, and a predictive power of *R*^2^ = 0.79, better than PS value. In this model, the only significant linear factor was the flow (*p* > 0.05); on the quadratic terms, only CaCl_2_ concentration showed significance (*p* > 0.05), and concerning to interactions, flow in interaction with CaCl_2_ concentration was significant (*p* > 0.05). The above indicates that the CaCl_2_ concentration combined with flow are the most influential factors for CV, most probably due to crystals forming.

**Figure 5:**
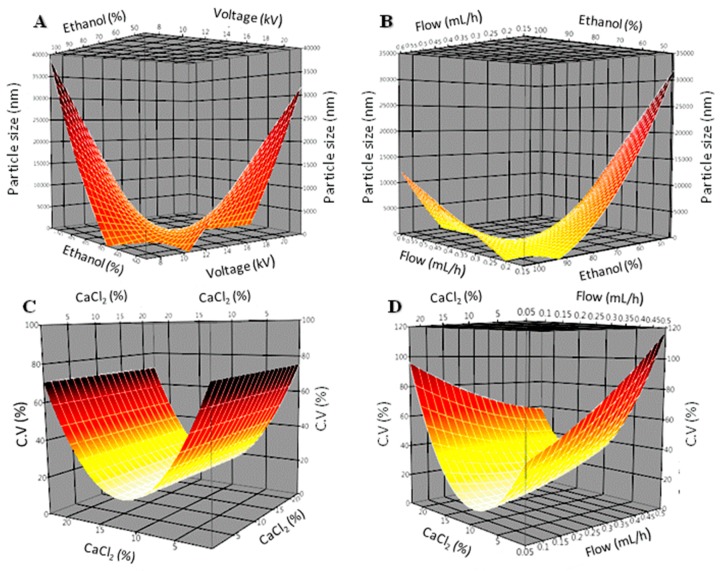
Response surface plots showing the most significant interactions. Ethanol conc. vs. voltage, and flow vs. ethanol conc. for size particle (**A**,**B**); CaCl_2_ quadratic effect, and CaCl_2_ vs. flow, for CV (**C**,**D**).

The interactive effects between factors and response to particle size and CV can be observed graphically in the response surface analysis (Figure 5). For clarity, only significant interactions are shown. Factors omitted in each graph were kept constant in their central values.

On particle size, the interaction between voltage and ethanol concentration (*p* = 0.0089) is observed in Figure 5A. With a saddle-type behavior, where the particle size increases or decreases from the center, two ascending curves arise, with a more pronounced curve toward the high values of voltage and flow. The region of smaller particle size value is situated at high concentrations of ethanol and flow from medium to low levels. This behavior clearly strengthens the hypothesis of the authors that ethanol presence would lower the crosslinking solution (cs) polarity and pectin’s solubility. As a result, little to non-aggregation was observed as well as pectin dispersion in the cs. Integrity of the particles was favored and even for low ethanol concentrations aggregation was eliminated. The role of ethanol and CaCl_2_ in the crosslinking solution is evident from surface analysis, where optimal ranges are noted along with flow values. As a result, the various combinations were set for the post-optimization assays.

On the other hand, CaCl_2_ concentration (*p* = 0.0001) has a positive effect on the CV (Figure 5C). The lowest CV values are among the levels 10% and 15% CaCl_2_ concentration, and higher levels of CV are found in the low and high extremes of CaCl_2_ concentration. Moreover, CaCl_2_ concentration vs. flow interaction show a positive effect (*p* = 0.0468). In Figure 5D, it was observed that the lowest values for CV are found at CaCl_2_ concentrations within 7% and 11% at low injection flow (0.05–0.1 mL/h). The particles size CV values increase up to 50% by combining high flow and low concentrations of CaCl_2_. The later observations confirm the role of CaCl_2_ in the stabilization of the surface of the particles; by meanly, reacting to pectin’s COO^−^ anionic groups, and thus preventing the shell to stablish links with other particles forming in the suspension. Nevertheless, too much CaCl_2_ in solution derives in nucleation of crystals and affects particle size assessment.

The optimal response to particle size and size CV was estimated by controlling the predictive profiles from the MSR tool. Evidently, more than one possible solution for the optimal desired response were found. Regarding particle size, the predictive profile estimated three different solutions for 1 μm diameter particles (Figure 5A,B). These profiles show a linear behavior for flow, voltage and CaCl_2_ concentration, and a quadratic effect for ethanol concentration. On counterpart, CV prediction profiles are displayed in Figure 5C,D. Two optimal solutions are observed for the lowest coefficient of variation possible. Besides voltage linear predictive behavior, all other factors show a quadratic effect, which gave stance to post-optimization tests. 

### 3.4. Post-Optimization Evaluation

**Figure 6:**
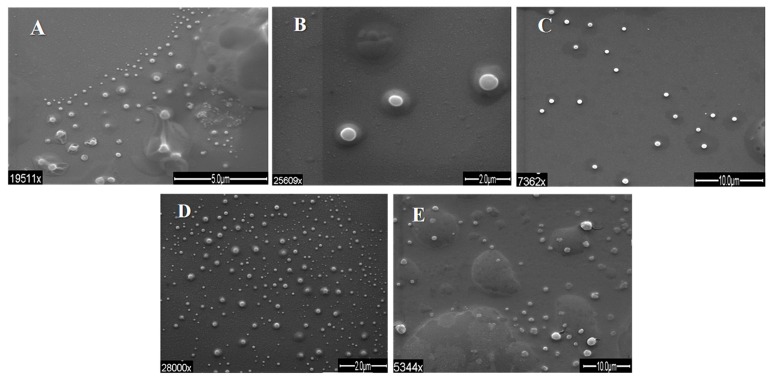
SEM images of post optimization beads morphology from samples (**A**–**E**) particles. Letters of images correspond to the same sample letter.

**Figure 7:**
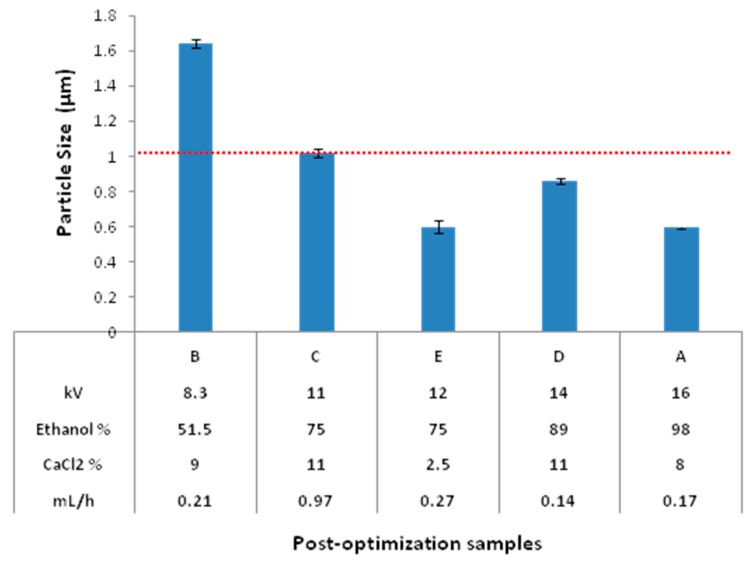
Mean diameters of post-optimization beads for each combination of post-optimization parameters. Error bars indicate standard error of the mean. Horizontal dotted red line indicates the optimal size (1 μm).

In order to compare the various sets of conditions generated by the statistical approach, a subsequent fabrication was carried out. The different sets of conditions for 1 μm particle size and CV inferred from our predictive profiles are shown in Table 1. Beads obtained according to the various sets of optimal conditions for 1 µm particle size and low CV from our predictive profiles were carried out in triplicate and analyzed by SEM. From the range of conditions found, five sets were tested, and the results are shown in Figure 6 and Figure 7. Figure 6 shows SEM images for each set, from which 6C outstands the others. Figure 7 shows particle size for each set, and confirms the uniformity of the particles obtained by electrospray. Fine, round, single spherical structures with no presence of aggregates were observed. In addition, the interference caused by the calcium was removed, validating the effectiveness of a “wash” process. The latter allowed using image processing software to obtain numerical data on beads diameter.

With the data obtained for diameter measurements, central tendency parameters (mean, median, and mode) and the standard error for each sample were obtained. In general, samples were adjusted very close to the expected values of about 1 μm, with less variation, as seen in the standard error. The results confirm that our model for optimization of the manufacturing process was effective.

In Figure 7, the average particle size values for each set of conditions are presented. The two most significant factors (voltage and ethanol concentration) are in ascending magnitude order; a tendency in this order is evident for both factors in an inverse relationship. The lowest values tested generated the highest particle size. This behavior can be explained by the effect of a low voltage in the formation of a less definite Taylor cone, resulting in bigger droplets. Similarly, the effect of solvent polarity to maintain droplets separate in the crosslinking solution is lessened when the ethanol concentration was lower.

With the results found, it may be hypothesized that the high variation in particle size found in the former analysis by DLS could had been due to interference from high concentrations of calcium chloride present in the samples, causing nucleation in particle surface; nevertheless, the latter do not invalid effect caused by the conditions tested. Also, high concentrations of CaCl_2_ could generate excessive interference by crystal nucleation on the particles surface, which was eliminated by a “wash” process.

Optimization of the manufacturing process has been successfully completed with the optimal assay conditions (Figure 7C), for particles with average size of 1.02 ± 0.024 μm.

## 4. Conclusions

The combined gel features of DTA pectin and AXF from maize allowed fast formation without aggregation of insulin-loaded beads through coaxial-electrospray. The optimization of the manufacturing factors for beads with an average size of 1 μm were 11 KV and 0.972 mL/h, with a reception crosslinking solution of 11% of CaCl_2_ in ethanol 75%. The above values are the result of a statistical analysis of surface response methodology, where a mathematical model with a predictive power of 70% was obtained.

The beads showed a core with insulin and arabinoxylans and, apparently, a shell of pectin. Beads produced under optimal conditions were symmetrical and stable without aggregation, with an average particle size of 1.02 ± 0.24 μm. All were stable with no aggregation surface particles. 

Due to their structure, composition, and size, DTA pectin/AXF beads are promising for future testing on degradation and insulin release under human gastrointestinal tract simulated conditions. Further research is recommended for in vivo evaluations on glycemic control in animal models. The coaxial-electrospray technique used in this work is a semi-automated and industrial-suitable method. This latter feature favors the social appropriation of the results of this work in the middle term.

## Figures and Tables

**Table 1:** Set of conditions from the statistical analysis.

Run	Voltage (KV)	Flow (mL/h)	Ethanol Conc. (%)	CaCl_2_ Conc. (%)
A	16	0.175	98	8
B	8.3	0.208	51.5	9
C	11	0.972	75	11
D	14.5	0.142	89.5	11
E	12	0.275	75	2.5
